# Evaluation of Neurotoxicity of NBOH Derivatives

**DOI:** 10.3390/ph19071055

**Published:** 2026-07-08

**Authors:** Rodrigo Foss da Silva, Lennon Machado Alves, Fernanda Mocellin Conte, Bruno Pereira dos Santos, Rodrigo Ligabue-Braun, Solange Cristina Garcia, Tiago Franco de Oliveira, Hecson Jesser Segat, Silvana Peterini Boeira, Dieniffer Espinosa Janner, Ana Cristina Correa Carvalhal Ferreira, Ariane Correa Carvalhal Ferreira, Marcelo Dutra Arbo

**Affiliations:** 1 Faculdade de Farmácia, Universidade Federal do Rio Grande do Sul, Porto Alegre 90610-000, RS, Brazil; rodrigofoss53@gmail.com (R.F.d.S.); lennon.machado.a@gmail.com (L.M.A.); fconte@universo.univates.br (F.M.C.); solangecgarcia88@gmail.com (S.C.G.); 2 Departamento de Farmacociências, Universidade Federal de Ciências da Saúde de Porto Alegre, Rua Sarmento Leite, 245, Porto Alegre 90050-170, RS, Brazil; brunosan777@gmail.com (B.P.d.S.); rodrigolb@ufcspa.edu.br (R.L.-B.); oliveira@ufcspa.edu.br (T.F.d.O.); 3 Programa de Pós-Graduação em Bioquímica, Universidade Federal do Pampa, Uruguaiana 97500-970, RS, Brazil; hecsonsegat@unipampa.edu.br (H.J.S.); silvanaboeira@unipampa.edu.br (S.P.B.); dienifferjanner.aluno@unipampa.edu.br (D.E.J.); 4 Campus Itaqui, Universidade Federal do Pampa, Itaqui 97650-000, RS, Brazil; anacarvalhal.aluno@unipampa.edu.br (A.C.C.C.F.); arianeferreira.aluno@unipampa.edu.br (A.C.C.F.)

**Keywords:** NBOHs, neurotoxicity, SH-SY5Y, mitochondrial membrane potential

## Abstract

**Background/Objectives**: The study sought to investigate the neurotoxicity of NBOHs, which are a class of new psychoactive substances (NPSs) that act as agonists of serotonin 5-HT2A and 5-HT2C receptors, resulting in hallucinogenic effects similar to those of LSD, often found in paper blotters. **Methods**: Differentiated SH-SY5Y cells were incubated for 24 h with 0.5, 1.0, 3.5, 5 and 10 nM of the derivatives 25I-NBOH, 25E-NBOH and 25B-NBOH. Cell viability was assessed by MTT reduction and neutral red uptake assays. Mitochondrial membrane potential and production of free radicals were also evaluated. In addition, *Drosophila melanogaster* flies were exposed to 5, 10 and 50 nM of each NBOH derivative for 4 h. Negative geotaxis, CAT, and AChE enzymatic activities were evaluated. **Results**: Both 25E-NBOH and 25B-NBOH decreased cell viability. Furthermore, mitochondrial hyperpolarization of cells and an increase in oxidative species were observed in response to higher concentrations of 25E-NBOH and 25B-NBOH. In *D. melanogaster*, 25B-NBOH increased climbing time and both 25E-NBOH and 25B-NBOH increased CAT and AChE activities at 50 nM. **Conclusions**: These findings indicate greater toxicity of the 25E- and 25B-NBOH compared to 25I-NBOH, suggesting metabolic activity or mitochondrial signaling, oxidative stress, and cholinergic dysregulation as a potential mechanism related to NBOH neurotoxicity.

## 1. Introduction

In recent decades, the increase in the availability and consumption of a variety of psychoactive substances, often known as new psychoactive substances (NPSs), has posed a growing threat to public health [1]. NPSs are predominantly composed of molecules developed for illicit purposes, aiming to circumvent national and international control measures applied to already regulated substances, whose effects they seek to imitate or reproduce. These exhibit effects similar to those of other widely known and prohibited drugs.

Between 2009 and 2017, the European Monitoring Center for Drugs and Drug Addiction [1] reported 802 NPSs in more than 111 countries, of which 136 were phenethylamine derivatives, including NBOMes (25I-NBOMe) and NBOHs (25I-NBOH). By the end of 2021, the EMCDDA was monitoring around 880 new psychoactive substances, of which 52 were reported for the first time in Europe in 2021.

The pharmacological and toxicological characteristics of NPSs differ greatly from each other. They are generally divided into four main classes: cannabinoids, psychostimulants, opioids and hallucinogens [2]. Within this group, the phenethylamines, to which the NBOMe and NBOH classes belong, stand out in the hallucinogens category. 25I-NBOMe [2-(4-iodo-2,5-dimethoxyphenyl)-N-ethanamine] was the first of the NBOMe series to emerge, followed later by the development and launch of other NBOMes on the illegal market [3].

Both NBOMes and NBOHs are serotonin 5-HT2A and 5-HT2C receptor agonists, resulting in hallucinogenic effects similar to those of LSD. Effects include alterations in sensory perception, thinking, emotions, and consciousness, with visual and auditory hallucinations, mood changes, and altered time perception, among other psychoactive effects [3,4,5].

**Figure 1:**
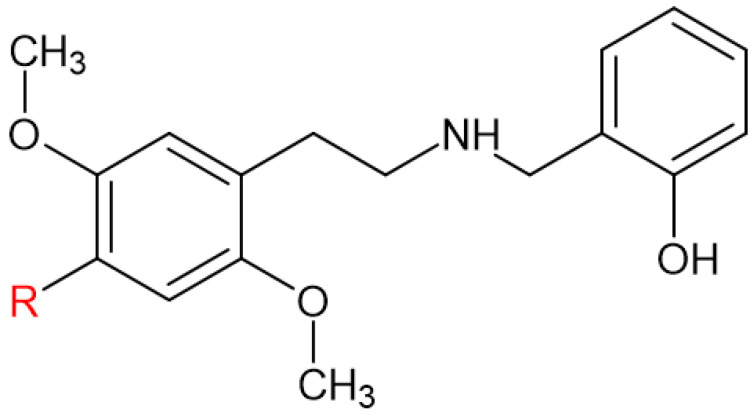
Chemical structure of NBOH derivatives, if R is (I) 25I-NBOH, (Ethyl) 25E-NBOH, (Br) 25B-NBOH.

NBOHs (Figure 1) are often found in the form of stamps, which is a common form of delivery for psychoactive substances. The stamps are found in the form of blotting paper, which is an absorbent paper matrix that can be impregnated with chemicals, allowing for controlled dosing and administration [6]. This form of delivery is convenient for both dealers and users, as blotting paper can be easily transported and concealed, and allows for precise administration of the substances.

Due to the relatively new nature of these substances, much remains to be understood about their long-term effects and abuse potential. Therefore, it is critical that more research be conducted to adequately assess the risks associated with the use of NBOHs [7]. Currently, there are no studies available on the neurotoxicity of these substances. Therefore, the purpose of this study was to investigate the in vitro neurotoxicity of NBOH-derived drugs using the differentiated human neuroblastoma cell line SH-SY5Y.

## 2. Results

### 2.1. NBOH Derivatives Reduced Cell Viability in Differentiated SH-SY5Y Cells

**Figure 2:**
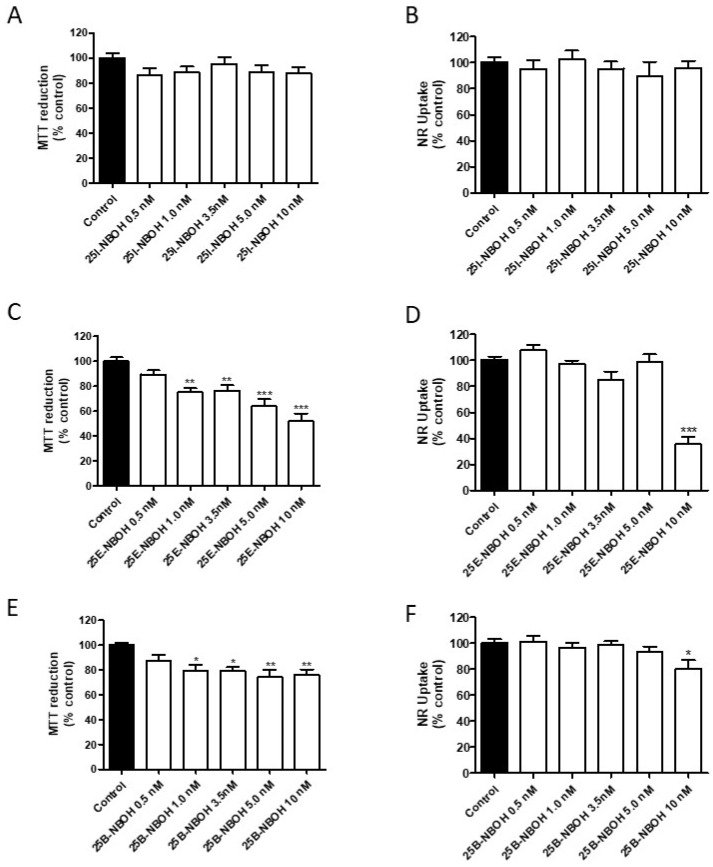
Cytotoxicity presented by 25I-NBOH (**A**,**B**), 25E-NBOH (**C**,**D**) and 25B-NBOH (**E**,**F**) in differentiated SH-SY5Y cells evaluated by MTT reduction (**A**,**C**,**E**) and NR uptake (**B**,**D**,**F**) assays after 24 h incubation. Data are presented as percentage of cell death relative to the negative controls. Three independent experiments were performed with each concentration tested in three replicates within each experiment. Statistical comparisons were made using one-way ANOVA/Bonferroni post hoc test (* *p* < 0.05; ** *p* < 0.01; *** *p* < 0.001 vs. control).

The cytotoxicity was carried out after incubation of the differentiated SH-SY5Y cells with 0–10 nM of each NBOH for 24 h and evaluated by the MTT reduction (Figure 2A,C,E) and NR uptake (Figure 2B,D,F) assays. 25I-NBOH did not cause a reduction in cell viability at the tested concentrations in both MTT redution (Figure 2A) and NR uptake (Figure 2B) assays. However, a statistically significant (*p* < 0.05, ANOVA/Bonferroni) reduction in cell viability was observed for 25E-NBOH (Figure 2C,D) and 25B-NBOH (Figure 2E,F) at 1.0, 3.5, 5.0, and 10 nM for MTT reduction assay and at 10 nM for NR uptake assay. Among all NBOH derivatives, 25E-NBOH seems to be the most potent.

### 2.2. NBOH Derivatives Induced Mitochondrial Hyperpolarization in Differentiated SH-SY5Y Cells

**Figure 3:**
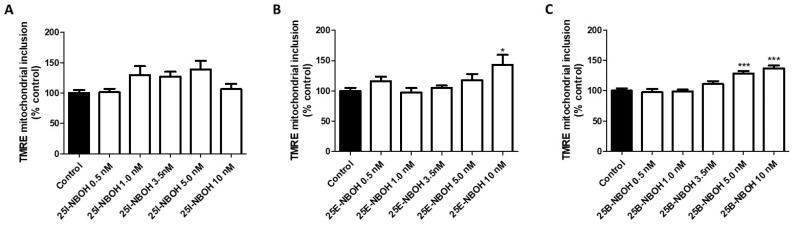
Mitocondrial membrane potential measured in differentiated SH-SY5Y cells after 24 h incubation with 25I-NBOH (**A**), 25E-NBOH (**B**), and 25B-NBOH (**C**) derivatives at 37 °C. Results are expressed as percentage control ± SEM (n = 3 independent experiments run in three replicates). Statistical comparisons were made using one-way ANOVA/Bonferroni post hoc test (* *p* < 0.05; *** *p* < 0.001 vs. control).

As shown in Figure 3A, 25I-NBOH did not induce any alteration in the mitochondrial membrane potential. However, 25E-NBOH (Figure 3B) significantly increased the mitochondrial membrane potential at 10 nM (*p* < 0.05, ANOVA/Bonferroni), while 25B-NBOH (Figure 3C) significantly hyperpolarized mitochondria at 5.0 and 10 nM (*p* < 0.001, ANOVA/Bonferroni).

### 2.3. NBOH Derivatives Increased the Production of Reactive Species in Differentiated SH-SY5Y Cells

**Figure 4:**
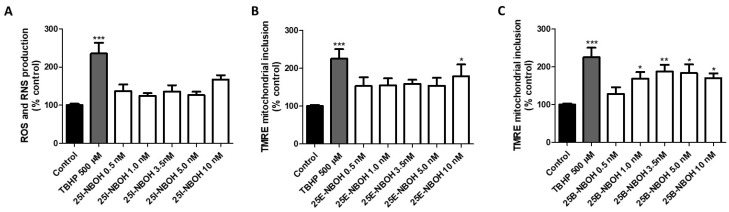
Reactive species measured in differentiated SH-SY5Y cells after 24 h incubation with (**A**) 25I-NBOH, (**B**) 25E-NBOH and (**C**) 25B-NBOH derivatives at 37 °C. Results are expressed as percentage control ± SEM (n = 3 independent experiments run in three replicates). Statistical comparisons were made using one-way ANOVA/Bonferroni post hoc test (* *p* < 0.05; ** *p* < 0.01; *** *p* < 0.001 vs. control).

Figure 4 depicts the results in the production of reactive species. 25I-NBOH did not induce any alteration in the reactive species formation (Figure 4A). However, 25E-NBOH (Figure 4B) significantly increased the formation of reactive species at 10 nM (*p* < 0.05, ANOVA/Bonferroni), while 25B-NBOH (Figure 4C) significantly increased the formation of reactive species at 1, 3.5, 5.0 and 10 nM (*p* < 0.05, ANOVA/Bonferroni).

### 2.4. NBOH Derivatives Alter Locomotor Activity in D. melanogaster

**Figure 5:**
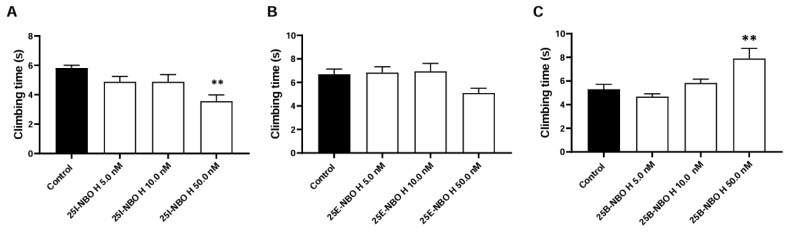
Climbing time obtained from *Drosophila melanogaster* exposed to (**A**) 25I-NBOH, (**B**) 25E-NBOH and (**C**) 25B-NBOH derivatives. Data are expressed as mean ± SEM (n = 5). Statistical comparisons were made using one-way ANOVA/Bonferroni post hoc test (** *p* < 0.01 vs. control).

A significant reduction (*p* < 0.01, ANOVA/Bonferroni) in climbing time in the 50 nM 25I-NBOH-treated group compared to the control group (Figure 5A); however, a significant increase (*p* < 0.01, ANOVA/Bonferroni) in this parameter was observed for 50 nM 25B-NBOH-treated flies (Figure 5C). For 25E-NBOH (Figure 5B), any significant alterations were observed 4 h after treatment.

### 2.5. NBOH Derivatives Modulate Enzymatic Activities

**Figure 6:**
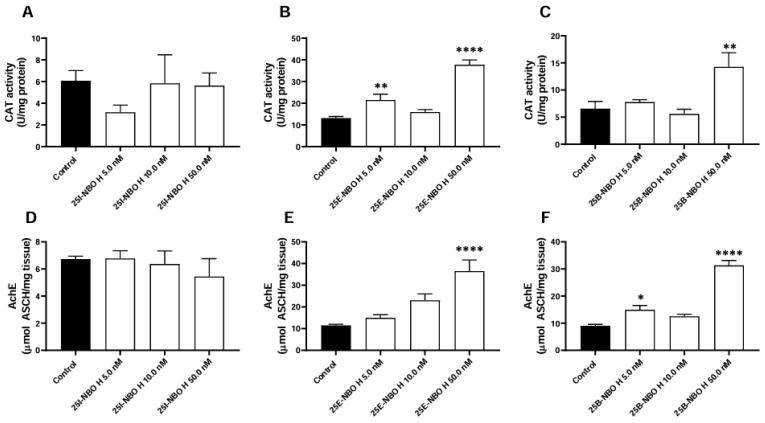
Catalase activity (CAT, (**A**–**C**)), and Acetylcholinesterase activity (AChE, (**D**–**F**)) of *Drosophila melanogaster* exposed to 25I-NBOH (**A**,**D**), 25E-NBOH (**B**,**E**), and 25B-NBOH (**C**,**F**) derivatives. Data are expressed as mean ± SEM (n = 5). Statistical comparisons were made using one-way ANOVA/Bonferroni *post hoc* test (* *p* < 0.05; ** *p* < 0.01; **** *p* < 0.0001 vs. control).

No significant differences were observed in CAT (Figure 6A) or AChE activity (Figure 6D) at any tested concentration of 25I-NBOH. However, CAT activity in the 25E-NBOH-exposed groups was increased at 5.0 nM (*p* < 0.01; ANOVA/Bonferroni) and 50 nM (*p* < 0.0001, ANOVA/Bonferroni) compared to the control group (Figure 6B). Regarding AChE activity, a significant increase was observed at 50 nM 25E-NBOH (*p* < 0.001; ANOVA/Bonferroni) (Figure 6E). In the 25B-NBOH-exposed groups, an increase in CAT activity was observed at 50 nM compared to the control group (*p* < 0.01; ANOVA/Bonferroni) (Figure 6C), without a clear dose-dependent pattern across concentrations. Additionally, AChE activity was increased at 5.0 nM (*p* < 0.05; ANOVA/Bonferroni) and 50.0 nM (*p* < 0.0001; ANOVA/Bonferroni) compared to the control group (Figure 6F), suggesting concentration-specific effects rather than a consistent dose–response relation-ship.

### 2.6. In Silico Prediction Points to Genotoxicity and Immunotoxicity

Different ADMETox aspects of NBOH derivatives were inspected by employing varied computational prediction tools. Overall confidence on the results is in the 70% range, with immunotoxicity, respiratory toxicity, and cytochrome 2D6 inhibition being more confidently predicted. The potential to disrupt neuroendocrine pathways is highlighted by predicted interactions with different neurotransmitter and hormonal receptors. Overall, 25B-NBOH, 25E-NBOH, and 25I-NBOH share a similar ADMETox profile. Full reports are shown in Supplementary File S1, while Table 1 summarizes the main findings.

## 3. Discussion

NBOH derivatives are a family of NPS that began to appear in blotters [8] seized in Brazil in 2014. In the last decade, the NBOHs are among the five most detected synthetic drugs in Brazil [9]. Poisoning cases involving NBOH compounds have been reported in recent years, evidencing the considerable toxicity of this drug [10]. Therefore, the knowledge of the mechanisms of toxicity of the NBOHs represents a great advance in the diagnosis and treatment of such cases. The in silico prediction of potential toxicity targets could not differentiate clearly among the three inspected molecules, reinforcing the need for in-depth experimental investigations on the different toxicity profiles for these compounds.

Regarding the tested substances, 25I-NBOH does not seem to exert significant effects on the SH-SY5Y viability at the concentrations tested, proving to be the least potent NBOH derivative. According to our results, in vivo experiments have pointed out that 25I-NBOH may be less liable to illicit use [11]. This may be crucial information for future studies exploring the therapeutic or adverse potential of this substance in neurobiological contexts. However, 25E-NBOH and 25B-NBOH negatively impact the differentiated SH-SY5Y cell viability. Interestingly, in a recent poisoning case described in Brazil, a male teenager consumed a blotter paper sold as LSD and hours later he was found dead. The toxicological analysis indicated the presence of 25E-NBOH in the organism, evidencing the considerable toxicity of this drug [12]. Overall, this difference in the cytotoxicity of the NBOH derivatives highlights the importance of considering structural variations in related compounds.

Interestingly, the neurotoxicity of 25H-NBOH, another derivative, was investigated in an ex vivo organotypic culture model of hippocampal slices, showing a progressive neuron loss even after seven days of removal of the drug from cell culture medium. In addition, 25H-NBOH also induced progenitor cell differentiation and increased the density of post-mitotic neurons [13].

A difference in cytotoxicity was observed comparing the cell death assays applied in this study. NR uptake assay showed a significant reduction only at a concentration of 10 nM, while the MTT reduction revealed a reduction in cell viability at lower concentrations. These discrepancies suggest that the sensitivity of the assays may vary in response to the specific characteristics of each substance. Mainly, the MTT reduction assay measures the activity of cellular dehydrogenases, while NR uptake is based on the storage of the dye in lysosomes and the Golgi complex of viable cells [14]. In toxicological studies, this is quite common [15,16,17,18,19] due to the different endpoints measured in each assay. In addition, chemical compounds can stimulate metabolic activity in a cell; dehydrogenases are sensitive to changes in the concentration and flux of ions in their environment. Thus, the activity of these enzymes can be increased by appropriate ionic conditions [20]. Therefore, the choice of the method for assessing cell viability not only influences the interpretation of the results but also highlights the importance of selecting the most sensitive approach to detect the specific effects of drugs. However, it is important to note that MTT reduction and neutral red uptake assays provide indirect measures of cell viability and cellular function and do not allow discrimination between different modes of cell death.

On mitochondrial activity, as expected based on cytotoxicity results, 25I-NBOH did not induce any significant change. On the other hand, when evaluating 25E-NBOH and 25B-NBOH, significant mitochondrial hyperpolarization was observed. This hyperpolarization may suggest a specific response of the mitochondria to higher concentrations of these substances, indicating potential modifications in metabolic activity or mitochondrial signaling, which should be investigated in depth in future studies. A positive correlation was established between mitochondrial membrane potential and neuronal survival, with neurons displaying a more pronounced hyperpolarization surviving longer [21]. This hyperpolarization can be seen as a tentative increase in ATP synthesis, which may, ultimately, lead to cell death by apoptosis. However, cell death mechanisms induced by NBOHs, such as apoptosis or necrosis, still deserve investigation.

One of the best described mechanisms underlying the toxicity of many xenobiotics is oxidative stress. Reactive species play a pivotal role in the cytotoxic effects of drugs such as amphetamine derivatives [22,23] and cocaine [24]. Our results showed that, similarly, 25E-NBOH and 25B-NBOH increased the production of reactive species while no alterations were observed for 25I-NBOH. In spite of the increase in production of free species itself not being sufficient to address the importance of the oxidative stress in the cytotoxic effect presented by the NBOHs, it is a clear indication that this pathway should be better investigated. In addition, it reinforces the effect of chemical structure in the toxicity played by the derivatives.

In *D. melanogaster*, the negative geotaxis assay was initially performed to assess locomotor behavior. Therefore, increased climbing time is indicative of impaired locomotor performance [25,26,27]. In contrast to 25B-NBOH, 25-I NBOH exposure reduced climbing time, indicating an altered locomotor response characterized by increased climbing activity. Similar alterations in locomotor behavior have been reported for psychoactive compounds, including amphetamine, in *D. melanogaster* [28]. Thus, the increased locomotor activity observed after 25I-NBOH exposure may reflect a change in behavioral state rather than a direct enhancement of motor performance. Considering that NBOH compounds, including 25I-NBOH, are potent serotonin 5-HT2A receptor agonists [3,29], serotonergic modulation may contribute to the behavioral effects observed; however, the specific neurobiological mechanisms underlying this response remain to be elucidated. Interestingly, 25B-NBOH, at the highest tested concentration, significantly increased climbing time, indicating impaired locomotor performance. This effect may be associated with alterations in motor coordination and neuromuscular function, which are commonly observed in models exposed to neuroactive or neurotoxic compounds [30]. Altogether, these findings suggest that structurally related NBOH derivatives can induce distinct behavioral responses, indicating differential biological effects, despite their chemical similarities.

Additionally, flies exposed to the 25E- and 25B-NBOH showed increased AChE activity at the highest concentration tested, whereas no such increase was observed in the 25I-NBOH-exposed group. AChE plays a key role in locomotor behavior by regulating cholinergic neurotransmission, which is essential for muscle contraction and motor coordination [31,32]. Therefore, increased AChE activity may result in reduced acetylcholine availability and altered cholinergic signaling, potentially affecting motor responses [31]. The concomitant increase in AChE activity and impaired locomotor performance observed after 25B-NBOH exposure suggests that alterations in cholinergic homeostasis may be associated with the behavioral effects induced by this compound. Nevertheless, additional investigations are necessary to determine whether these biochemical changes directly contribute to the observed locomotor alterations.

Finally, an increase in CAT activity at the highest tested concentration was observed in flies exposed to 25E- and 25B-NBOH, but not in those exposed to 25I-NBOH. CAT is an endogenous antioxidant enzyme responsible for the degradation of hydrogen peroxide into water and oxygen [33]. Therefore, increased CAT activity may indicate activation of antioxidant defenses in response to cellular stress or redox imbalance [34]. However, markers of oxidative damage such as lipoperoxidation and carbonyl protein content warrants further investigation in future studies. Taken together, the findings suggest that 25E- and 25B-NBOH may induce greater biological alterations compared with 25I-NBOH, involving changes in antioxidant responses and cholinergic signaling that are associated with locomotor impairment. These results highlight the importance of evaluating the toxicological profiles of emerging psychoactive substances, as structurally similar compounds may display distinct biological effects. Taken together, the in vitro and in vivo findings indicate greater toxicity of the 25E- and 25B-NBOH compared to 25I-NBOH, suggesting that cholinergic dysregulation and oxidative stress may underline the observed locomotor impairment.

Overall, these results provide additional information on the biological effects of NBOH derivatives in *D. melanogaster*, integrating behavioral and biochemical endpoints. Although these compounds are known 5-HT2A receptor agonists, data on their toxicolog-ical effects remain limited. In this study, 25E- and 25B-NBOH induced changes in loco-motor behavior, AChE, and CAT activity, while 25I-NBOH showed a distinct profile. The mechanisms underlying these effects remain unclear. These results add partial evidence to the current knowledge and support further investigation of this class of compounds.

Despite the novelty of this study in integrating in silico, in vitro, and in vivo approaches to investigate the mechanisms underlying the toxic effects of NBOHs, several limitations should be acknowledged. First, the in vitro experiments were conducted using a single cell model. The nervous system is a highly complex tissue in which neurons, astrocytes, microglia, and other cell types interact dynamically, and such interactions may significantly influence toxicological responses. In addition, the 24 h incubation protocol employed in the in vitro assays does not accurately reflect real-world patterns of NBOH use, where both dosage and duration of exposure are highly variable and difficult to standardize. Another limitation is the lack of more specific biomarkers to comprehensively evaluate certain toxicity mechanisms, which restricts a clearer understanding of the molecular pathways involved in the toxic effects of these compounds. Nevertheless, the findings obtained from the combined experimental approaches allowed the identification of potentially relevant pathways that may contribute to the knowledge of NBOH-induced toxicity. These pathways should be further explored in future studies to provide a more detailed mechanistic understanding of the toxicological effects associated with this class of psychoactive substances.

## 4. Materials and Methods

### 4.1. Chemicals

Dulbecco’s Modified Eagle’s Medium (DMEM) high glucose, trypsin (0.25%)-ethylenediaminetetracetic acid (EDTA) (1 mM), penicillin 10,000 UmL^−1^, streptomycin 10,000 μgmL^−1^, amphotericin B solution 250 μgmL^−1^, non-essential aminoacids (NEAAs), heat inactivated fetal bovine serum, 12-O-tetradecanoylphorbol-13-acetate (TPA), tetramethyl-rhodamine ethyl ester perchlorate (TMRE), 3-(4,5-dimethylthiazol-2-yl)-2,5-diphenyltetrazoliumbromide (MTT), 3-amino-7-dimethylamino-2-methylphenazinehydrochloride (neutral red dye), trypan blue, and triton x-100 were purchased from Sigma-Aldrich (St. Louis, MO, USA). Dichlorodihydrofluorescein diacetate (DCFH-DA) was purchased from Invitrogen (Waltham, MA, USA). Dimethylsulfoxide (DMSO) was obtained from Labsynth (Diadema, SP, Brazil). The 25B-NBOH, 25E-NBOH, and 25I-NBOH samples were obtained from forensic seizures analyzed by the Instituto-Geral de Perícias do Rio Grande do Sul (IGP-RS, Porto Alegre, Brazil). After completion of the forensic analyses, residual methanolic samples were concentrated under a nitrogen stream and reanalyzed at the Universidade Federal de Ciências da Saúde de Porto Alegre (UFCSPA). Compound identities were confirmed by LC–HRMS using a micrOTOF-Q III mass spectrometer (Bruker Daltonics, Bremen, Germany) coupled to a Prominence liquid chromatography system (Shimadzu, Kyoto, Japan), based on accurate mass measurements and the expected isotopic profiles. The concentrations of the NBOH compounds were determined by comparison with reference standards (Cerilliant^®^, Round Rock, TX, USA), and the experimentally determined concentrations were used to prepare the solutions employed in the assays. Examination of the chromatographic and full-scan mass-spectrometric profiles did not reveal major additional components under the analytical conditions employed. NBOH samples were stored at −20 °C.

Initially, 25I-NBOH was at a concentration of 0.6 µM, 25E-NBOH at 1.0 µM and 25B-NBOH at 10.5 µM in water. For the experiments, NBOH solutions were diluted in DMEM, at a concentration of 10 nM. Subsequently, from this solution, sequential dilutions of 5, 3.5, 1 and 0.5 nM were prepared on the day of the experiment. The concentrations tested in this study (1–10 nM) are physiologically relevant and were selected based on a forensic case reporting a blood concentration of 2.07 ng/mL of 25C-NBOMe [35], corresponding to approximately 6.16 nM. Although no human blood concentration data are currently available for NBOH compounds, the use of 25C-NBOMe as a reference was considered appropriate due to the structural similarity and comparable pharmacological and toxicological profiles shared by NBOMe and NBOH derivatives.

### 4.2. Cell Culture

SH-SY5Y cells were cultured in 75 cm^2^ flasks (Kasvi, Pinhais, PR, Brazil) at 37 °C in a humidified 5% CO_2_–95% air atmosphere using DMEM high glucose (4.5 gL^−1^), containing antibiotic (100 UmL^−1^ penicillin; 100 µgL^−1^ streptomycin), 2.5 mL^−1^ of NEAA, 2.4 gL^−1^ of sodium chloride (NaCl), 3.7 gL^−1^ of sodium bicarbonate (NaHCO_3_) and 10% previously inactivated fetal bovine serum (FBS). The incubation temperature was maintained at 37 °C in a humidified atmosphere with 95% air and 5% CO_2_. Cells were subcultured weekly by trypsinization (0.25% trypsin/EDTA) once they reached 80–90% confluency. The experiments were carried out between the 12th and 17th passages to avoid phenotypic changes. For differentiation, cells were plated at a density of 25,000 cellscm^−2^ in medium containing 10 μM retinoic acid and cultured at 37 °C. After 3 days, 80 nM TPA was added to the plates, and the cells were cultured for another 3 days at 37 °C. Stock solutions of retinoic acid (10 mM) and TPA (80 mM) were previously prepared in DMSO [18].

### 4.3. Cytotoxicity Assays

To evaluate cell viability, MTT reduction and neutral red (NR) uptake assays were used. MTT is converted by cellular dehydrogenases into formazan, an indicator of cellular metabolic activity [36]. NR is a weakly cationic dye, absorbed by the cytosol by non-ionic diffusion across the cell membrane to then accumulate in the lysosomes of viable cells [14].

#### 4.3.1. MTT Reduction Assay

After 6 days of differentiation, the culture medium was removed and the cells were incubated with 0.5, 1, 3.5, 5, and 10 nM of each NBOH derivative. As a negative control, cells were incubated with DMEM only. A total of 1% Triton x-100 in cell culture medium was used as positive control. After 24 h, the medium was removed and replaced with PBS containing 0.5 mgmL^−1^ MTT, followed by a 2 h incubation at 37 °C. After the incubation time, the solution was removed and then 100 µL of DMSO was added to each well to solubilize the formazan crystals. The absorbance was measured at 550 nm using a microplate reader (BioTek Synergy^®^ LX Multimode Reader, Winooski, VT, USA). Three independent experiments were performed, with each concentration analyzed in triplicate.

#### 4.3.2. NR Uptake Assay

After 6 days of differentiation, the culture medium was removed and the cells were incubated with 0.5, 1, 3.5, 5, and 10 nM of each NBOH derivative. As a negative control, cells were incubated with DMEM only. A total of 1% Triton x-100 in cell culture medium was used as positive control. After 24 h of incubation with NBOHs, the medium was removed and 200 uL of PBS containing 50 µgmL^−1^ of NR per well were added; subsequently, the cells were incubated at 37 °C for 3 h. Then, the wells were washed with 100 µL of PBS and 100 µL of lysis solution (50% ethanol: 1% glacial acetic acid in water) was added. Absorbance was quantified using a microplate reader (BioTek Synergy^®^ LX Multimode Reader, Winooski, VT, USA) set at 520 nm. Three independent experiments were conducted, with each concentration tested in triplicate.

### 4.4. Measurement of Mitochondrial Membrane Potential

TMRE is a positively charged compound that, after cellular permeation, accumulates in active mitochondria, which has a negative charge, in proportion to the mitochondrial membrane potential [37]. After 6 days of differentiation, the culture medium was removed and the cells were incubated with 0.5, 1, 3.5, 5, and 10 nM of each NBOH derivative at 37 °C. After 24 h incubation, the medium was replaced with PBS containing 2 µM TMRE and incubated for 30 min at 37 °C, protected from light. A stock solution of 2 mM TMRE in DMSO was prepared, stored at −20 °C, and protected from light. Fluorescence was measured at 37 °C on a microplate reader (BioTek Synergy^®^ LX Multimode Reader, Winooski, VT, USA) set to 544 nm excitation and 590 nm emission. Three independent experiments were conducted, with each concentration tested in triplicate.

### 4.5. Measurement of Intracellular Production of Reactive Species

The intracellular production of reactive oxygen (ROS) and nitrogen (RNS) species was measured using the dichlorodihydrofluorescein diacetate (DCFH-DA) assay as previously described [16]. Into the cells, intracellular esterases hydrolyze DCFH-DA to non-fluorescent dichlorodihydrofluorescein (DCFH), which will be oxidized by reactive species to form the fluorescent dichlorofluorescein (DCF). After differentiation, SH-SY5Y cells were washed with PBS and incubated with 10 μM DCFH-DA for 30 min at 37 °C, to promote the intracellular accumulation of the probe. Then, the cells were incubated with 0.5, 1, 3.5, 5, and 10 nM of each NBOH derivative. Tert-butylhydroperoxide (TBHP) 500 μM was used as a positive control. Fluorescence was set to 485 nm excitation and 530 nm emission and measured 24 h after incubation (BioTek Synergy^®^ LX Multimode Reader, Winooski, VT, USA). The data obtained were calculated as percentage of control from three independent experiments, with each concentration tested in three replicates within each experiment.

### 4.6. Drosophila Melanogaster Stock

Wild-type *Drosophila melanogaster* flies (Harwich strain) were used in this study. The flies were provided by the Laboratory of Pharmacological and Toxicological Evaluations Applied to Bioactive Molecules (LAFTAMBIO), Federal University of Pampa (Itaqui Campus, Rio Grande do Sul, Brazil). Flies were maintained under controlled conditions of temperature (25 ± 1 °C), relative humidity (60–70%), and a 12 h light/12 h dark photoperiod. The standard diet consisted of cornmeal, refined sugar, salt, whole milk powder, wheat germ, yeast, distilled water, and Nipagin^®^ (Dinâmica Química, Indaiatuba, SP, Brazil).

Flies were distributed into glass vials containing different experimental solutions. The groups were divided into a control group (fed exclusively with a 1% sucrose solution) and groups treated with 5, 10 and 50 nM of each NBOH derivative. Each drug was diluted in a 1% sucrose solution to ensure voluntary ingestion.

The flies were placed in glass vials containing filter paper soaked with the respective experimental solutions and were exposed for a period of 4 h. After this period of drug exposure, the flies were subjected to the behavioral tests described below and subsequently euthanized. For this experiment, 5 independent experiments were carried out (n = 5).

### 4.7. Negative Geotaxis

Locomotor performance was assessed using the negative geotaxis assay [38]. Flies were individually placed in graduated tubes (8 cm height) and allowed to acclimate for 1 min. They were then gently taped to the bottom, and the time required to reach the top was recorded, with a maximum cutoff of 120 s. Each fly underwent five trials, with five flies per group. Results were expressed in seconds.

### 4.8. Biochemical Analysis

For sample preparation, 30 fly heads were homogenized in 300 μL of 20 mM HEPES buffer (pH 7.0) and centrifuged at 78× *g* for 10 min. The supernatants were collected for enzymatic activity assays. Protein content was determined according to Bradford (1976) [39], using bovine serum albumin (BSA) as standard and Coomassie Brilliant Blue dye. Absorbance was measured at 595 nm using a microplate reader, with samples analyzed in duplicate. Results were used for normalization of biochemical data.

#### 4.8.1. Catalase (CAT) Activity

Catalase activity was determined as described by Aebi (1984) [40]. Briefly, 50 μL of supernatant was added to a cuvette containing 1 mL of reaction mixture (0.25 M potassium phosphate buffer, 2.5 mM EDTA, pH 7.0, 30% H_2_O_2_, and 10 μL Triton X-100). The decrease in absorbance was monitored at 240 nm for 2 min. CAT activity was expressed as U/mg of protein, and results represent the mean of seven independent experiments.

#### 4.8.2. Acetylcholinesterase (AChE) Activity

AChE activity was determined according to the method of Ellman et al. (1961) [41]. Samples containing 10 flies were homogenized in 400 μL of HEPES buffer and centrifuged at 1000× *g* for 10 min. The supernatant (20 μL) was incubated with reaction medium containing 0.1 M phosphate buffer (pH 8.0) and 5 mM DTNB, and the reaction was initiated by the addition of acetylthiocholine. Enzymatic activity was measured spectrophotometrically at 412 nm for 2 min and expressed as μmol of substrate hydrolyzed/h/mg of protein.

### 4.9. In Silico Predictions

Prediction of ADMETox properties (absorption, distribution, metabolism, excretion, and toxicity endpoints) of the molecules under study were performed with SwissADME [42], SuperPred 3.0 [43], Pred-hERG [44], Pred-Skin [45], eMolTOX [46], and ProTox3 [47].

### 4.10. Statistical Analysis

Statistical analysis was performed using GraphPad Prism software version 5.01 All results are expressed as percentage of control conditions and presented as mean ± SEM from three independent experiments. A Kolmogorov–Smirnov test was used to verify the normality of the data distribution. The comparisons between groups were performed with ANOVA, followed by a Bonferroni post hoc test. Significance was accepted at *p* < 0.05.

## 5. Conclusions

In conclusion, we describe, for the first time, the potential of 25I-NBOH and 25B-NBOH derivatives to induce neurotoxicity in differentiated SH-SY5Y cells and *D. melanogaster*. Among them, 25E-NBOH and 25B-NBOH were the most potent. Also, mitochondrial disturbances, characterized by hyperpolarization, were observed, as well as intracellular increase in reactive species production. The in vitro findings were corroborated in vivo, using *D. melanogaster*, and highlight the need to further investigate the molecular mechanisms underlying these responses, assessing more specific neurotoxicity and oxidative stress biomarkers, which will provide important information on the effects of NBOHs.

## Figures and Tables

**Table 1:** Summary of in silico toxicity endpoints with high confidence/probability (shown as percentage).

Endpoint	Compound		
	25E-NBOH	25B-NBOH	25I-NBOH
Respiratory toxicity	0.84	0.84	0.85
Immunotoxicity	0.97	0.99	0.99
Differential cytotoxicity	0.98	0.99	0.99
CYP2C19 Inhibitor	0.99	0.98	-
Activator of pregnane X receptor	0.99	-	-
Modulator of dopamine D1 receptor	0.98	0.98	0.98
Modulator of serotonin 2a receptor	-	0.99	0.99
Modulator of serotonin 2c receptor	-	0.99	0.99
Antagonist of the thyroid receptor	-	0.98	0.98
Proliferation of antigen-specific T-cells	0.96	0.60	0.60

## Data Availability

The original contributions presented in this study are included in the article/Supplementary Material. Further inquiries can be directed to the corresponding author(s).

## Supplementary Materials

The following supporting information can be downloaded at: https://www.mdpi.com/article/10.3390/ph19071055/s1, File S1: In silico data presented by NBOH derivatives.

## Abbreviations

The following abbreviations are used in this manuscript:

NPS	New psychoactive substance
EMCDDA	European Monitoring Center for Drugs and Drug Addiction
DMEM	Dulbecco’s Modified Eagle’s Medium
EDTA	Ethylenediaminetetracetic acid
PBS	Phosphate-buffer salt solution
NEAA	Non-essential aminoacid
MTT	3-(4,5-Dimethylthiazol-2-yl)-2,5-diphenyltetrazoliumbromide
NR	Neutral red
DMSO	Dimethylsulfoxide
TPA	12-O-Tetradecanoylphorbol-13-acetate
TMRE	Tetramethyl-rhodamine ethyl ester perchlorate
ROS	Reactive oxygen species
RNS	Reactive nitrogen species
DCFH-DA	Dichlorodihydrofluorescein diacetate
DCFH	Dichlorodihydrofluorescein
DCF	Dichlorofluorescein
TBHP	Tert-butylhydroperoxide
