# Supplementary material for: Evaluation of Neurotoxicity of NBOH Derivatives
Source: Pharmaceuticals (Basel). 2026 Jul 8;19(7):1055. doi: 10.3390/ph19071055 (PMC13414775; doi:10.3390/ph19071055)
Supplement: Supplementary file 1 [file pharmaceuticals-19-01055-s001.zip › pharmaceuticals-4339367-File S1.pdf]

25E-NBOH

Target Name	ChEMBL-ID	UniProt ID	PDB Visualization	TTD ID	Probability	Model accuracy
DNA-(apurinic or apyrimidinic site) lyase	CHEMBL5619	P27695	6BOW	T13348	98.08%	91.11%
Nuclear factor NF-kappa-B p105 subunit	CHEMBL3251	P19838	1SVC	*Not Available*	95.56%	96.09%
Cathepsin D	CHEMBL2581	P07339	4OD9	T67102	95.31%	98.95%
Transcription intermediary factor 1-alpha	CHEMBL3108638	O15164	4YBM	*Not Available*	91.68%	95.56%
Kruppel-like factor 5	CHEMBL1293249	Q13887	*Not Available*	*Not Available*	91.61%	86.33%
Endoplasmic reticulum-associated amyloid beta-peptide-binding protein	CHEMBL4159	Q99714	2O23	*Not Available*	91.52%	70.16%
Proteasome component C5	CHEMBL4208	P20618	6KWY	*Not Available*	91.31%	90%
Glucose transporter	CHEMBL2535	P11166	6THA	*Not Available*	90.16%	98.75%
Arachidonate 12-lipoxygenase	CHEMBL3687	P18054	3D3L	*Not Available*	87.8%	75.57%
Glycine transporter 2	CHEMBL3060	Q9Y345	*Not Available*	*Not Available*	87.73%	99.17%

Molecule 1

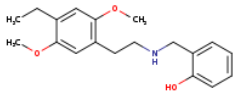

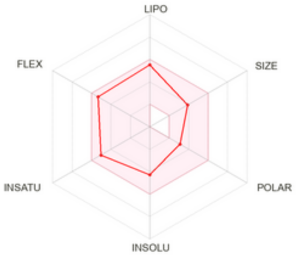

SMILES COc1cc(CC)c(cc1CCNCc1ccccc1O)OC

Physicochemical Properties	
Formula	C19H25NO3
Molecular weight	315.41 g/mol
Num. heavy atoms	23
Num. arom. heavy atoms	12
Fraction Csp3	0.37
Num. rotatable bonds	8
Num. H-bond acceptors	4
Num. H-bond donors	2
Molar Refractivity	93.09
TPSA	50.72 Å²

Lipophilicity	
Log $P_{o/w}$ (iLOGP)	3.57
Log $P_{o/w}$ (XLOGP3)	4.18
Log $P_{o/w}$ (WLOGP)	3.15
Log $P_{o/w}$ (MLOGP)	2.70
Log $P_{o/w}$ (SILICOS-IT)	4.31
Consensus Log $P_{o/w}$	3.58

Water Solubility	
Log *S* (ESOL)	-4.29
Solubility	1.63e-02 mg/ml ; 5.16e-05 mol/l
Class	Moderately soluble
Log *S* (Ali)	-4.95
Solubility	3.51e-03 mg/ml ; 1.11e-05 mol/l
Class	Moderately soluble
Log *S* (SILICOS-IT)	-6.63
Solubility	7.33e-05 mg/ml ; 2.32e-07 mol/l
Class	Poorly soluble

Pharmacokinetics	
GI absorption	High
BBB permeant	Yes
P-gp substrate	No
CYP1A2 inhibitor	Yes
CYP2C19 inhibitor	Yes
CYP2C9 inhibitor	No
CYP2D6 inhibitor	Yes
CYP3A4 inhibitor	Yes
Log K_p (skin permeation)	-5.26 cm/s

Druglikeness	
Lipinski	Yes; 0 violation
Ghose	Yes
Veber	Yes
Egan	Yes
Muegge	Yes
Bioavailability Score	0.55

Medicinal Chemistry	
PAINS	1 alert: mannich_A
Brenk	0 alert
Leadlikeness	No; 2 violations: Rotors>7, XLOGP3>3.5
Synthetic accessibility	2.28

25E-NBOH

Oral toxicity prediction results for input compound

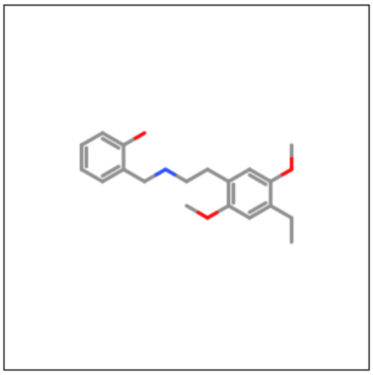

Predicted LD50: 300mg/kg

Predicted Toxicity Class: 3

1	2	3	4	5	6

Average similarity: 72.77%

Prediction accuracy: 69.26%

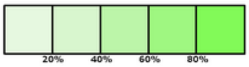

Name	CCC1=CC(=C(C=C1OC)(
Molweight	315.41
Number of hydrogen bond acceptors	4
Number of hydrogen bond donors	2
Number of atoms	23
Number of bonds	24
Number of rotable bonds	8
Molecular refractivity	93.09
Topological Polar Surface Area	50.72
octanol/water partition coefficient(logP)	3.7

Classification	Target	Shorthand	Prediction	Probability
Organ toxicity	Hepatotoxicity	dili	Inactive	0.77
Organ toxicity	Neurotoxicity	neuro	Active	0.59
Organ toxicity	Nephrotoxicity	nephro	Inactive	0.52
Organ toxicity	Respiratory toxicity	respi	Active	0.84
Organ toxicity	Cardiotoxicity	cardio	Inactive	0.59
Toxicity end points	Carcinogenicity	carcino	Inactive	0.64
Toxicity end points	Immunotoxicity	immuno	Active	0.97
Toxicity end points	Mutagenicity	mutagen	Inactive	0.73
Toxicity end points	Cytotoxicity	cyto	Inactive	0.74
Toxicity end points	BBB-barrier	bbb	Active	0.59
Toxicity end points	Ecotoxicity	eco	Inactive	0.52
Toxicity end points	Clinical toxicity	clinical	Inactive	0.51
Toxicity end points	Nutritional toxicity	nutri	Inactive	0.70
Tox21-Nuclear receptor signalling pathways	Aryl hydrocarbon Receptor (AhR)	nr_ahr	Inactive	0.91
Tox21-Nuclear receptor signalling pathways	Androgen Receptor (AR)	nr_ar	Inactive	0.96
Tox21-Nuclear receptor signalling pathways	Androgen Receptor Ligand Binding Domain (AR-LBD)	nr_ar_lbd	Inactive	0.98
Tox21-Nuclear receptor signalling pathways	Aromatase	nr_aromatase	Inactive	0.91
Tox21-Nuclear receptor signalling pathways	Estrogen Receptor Alpha (ER)	nr_er	Inactive	0.91
Tox21-Nuclear receptor signalling pathways	Estrogen Receptor Ligand Binding Domain (ER-LBD)	nr_er_lbd	Inactive	0.96
Tox21-Nuclear receptor signalling pathways	Peroxisome Proliferator Activated Receptor Gamma (PPAR-Gamma)	nr_ppar_gamma	Inactive	0.97
Tox21-Stress response pathways	Nuclear factor (erythroid-derived 2)-like 2/antioxidant responsive element (nrf2/ARE)	sr_are	Inactive	0.96
Tox21-Stress response pathways	Heat shock factor response element (HSE)	sr_hse	Inactive	0.96
Tox21-Stress response pathways	Mitochondrial Membrane Potential (MMP)	sr_mmp	Inactive	0.89
Tox21-Stress response pathways	Phosphoprotein (Tumor Supressor) p53	sr_p53	Inactive	0.95
Tox21-Stress response pathways	ATPase family AAA domain-containing protein 5 (ATAD5)	sr_atad5	Inactive	0.96
Molecular Initiating Events	Thyroid hormone receptor alpha (THRα)	mie_thr_alpha	Inactive	0.90
Molecular Initiating Events	Thyroid hormone receptor beta (THRβ)	mie_thr_beta	Inactive	0.78
Molecular Initiating Events	Transtyretrin (TTR)	mie_ttr	Inactive	0.97
Molecular Initiating Events	Ryanodine receptor (RYR)	mie_ryr	Inactive	0.98
Molecular Initiating Events	GABA receptor (GABAR)	mie_gabar	Inactive	0.96
Molecular Initiating Events	Glutamate N-methyl-D-aspartate receptor (NMDAR)	mie_nmdar	Inactive	0.92
Molecular Initiating Events	alpha-amino-3-hydroxy-5-methyl-4-isoxazolepropionate receptor (AMPAR)	mie_ampar	Inactive	0.97
Molecular Initiating Events	Kainate receptor (KAR)	mie_kar	Inactive	0.99
Molecular Initiating Events	Achetylcholinesterase (AChE)	mie_ache	Inactive	0.77
Molecular Initiating Events	Constitutive androstane receptor (CAR)	mie_car	Inactive	0.98
Molecular Initiating Events	Pregnane X receptor (PXR)	mie_pxr	Inactive	0.92
Molecular Initiating Events	NADH-quinone oxidoreductase (NADHOX)	mie_nadhox	Inactive	0.97
Molecular Initiating Events	Voltage gated sodium channel (VGSC)	mie_vgsc	Inactive	0.95
Molecular Initiating Events	Na+/I- symporter (NIS)	mie_nis	Inactive	0.98
Metabolism	Cytochrome CYP1A2	CYP1A2	Inactive	0.66
Metabolism	Cytochrome CYP2C19	CYP2C19	Inactive	0.63
Metabolism	Cytochrome CYP2C9	CYP2C9	Inactive	0.69
Metabolism	Cytochrome CYP2D6	CYP2D6	Active	0.74
Metabolism	Cytochrome CYP3A4	CYP3A4	Inactive	0.69
Metabolism	Cytochrome CYP2E1	CYP2E1	Inactive	0.97

25E-NBOH

Potential Toxicity:

Index	Action	Injury	Confidence	Similar active compound in database
000	Differential cytotoxicity (isogenic chicken DT40 Rev3 mutant cell line)	Genotoxicity	0.983	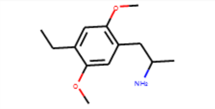
001	Inhibit CYP2C19 Activity	Liver	0.991	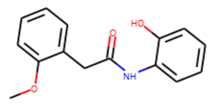
002	Activators of the human pregnane X receptor (PXR) signaling pathway	Liver	0.988	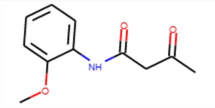
003	Modulator of Dopamine D1 receptor	Central nervous system, Kidney, Heart	0.984	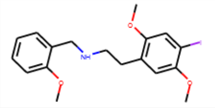
004	Differential cytotoxicity (isogenic chicken DT40 cell lines)	Genotoxicity	0.994	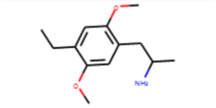
005	Differential cytotoxicity against isogenic chicken DT40 cell lines with known DNA damage response pathways - Rad54/ Ku70 mutant cell line	Genotoxicity	0.992	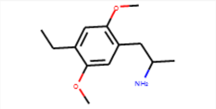

**Chemical Exposure**	**Molecular initiating event** *in chemico*	**Cellular response** *in vitro*		**Tissue / Organ response** *in vivo*	**Organism response** *in vivo*	**Pred-Skin 3.0 Outcome** *in silico*
•Skin Penetration •Electrophilic substance: directly or via auto-oxidation or metabolism	Covalent interaction with proteins in the skin (OECD442C) Haptenation: covalent modification of epidermal proteins	Keratinocyte responses (OECD442D) • Activation of inflammatory cytokines •Induce cytoprotective genes	Dendritic cells (DCs) (OECD442E) • Induction of inflammatory cytokines •Mobilization of DCs	Proliferation of antigen-specific T cells (OECD429) •Histocompatibility complex representation by DCs •Activation of T cells •Proliferation of activated T cells	Inflammation upon challenge allergen To maximise the use of existing knowledge, we also incorporate historical HRIPT (human repeated insult patch test) and HMT (human maximization test)	The Bayesian model is a consensus model integrating predictions from all the other assays for an integrative qualitative risk assessment (QRA) of skin sensitization based on the weight of evidence (WoE).
•Exposure consideration ? •Physicochemical and Biopharmaceutical properties ? •Skin Penetration ? •Skin Metabolism ? 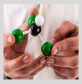	**Prediction DPRA** Non-Sensitizer (-) (AD, Confiability) (Outside , 69.3%) Probability map 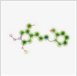	**Prediction KeratinoSens** Non-Sensitizer (-) (AD, Confiability) (Outside, 92.2%) Probability map 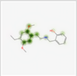	**Prediction h-CLAT** Non-Sensitizer (-) (AD, Confiability) (Outside, 62.6%) Probability map 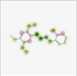	**Prediction LLNA** Sensitizer (+) (AD, Confiability) (Outside, 96.2%) Probability map 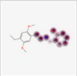	**Prediction HRIPT/HMT** Non-Sensitizer (-) (AD, Confiability) (Outside, 98.6%) Probability map 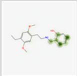	**Bayesian Outcome** Non-sensitizer (-) (Confiability) (High)

Low (-) confidence prediction for the Bayesian model means two or more individual predictions are in disagreement with Bayesian Outcome.

SMILES	Consensus Weighted	Binary Prediction	Confiabilty %	Applicability Domain	Fragment Contribution Maps and Explainable AI (XAI) for the Binary Model	Multiclass Prediction	Confiabilty %	Applicability Domain	Reg. prediction (pIC50)	Applicability Domain	Fragment Contribution Maps for the Regression Model
CCC1C(OC)=CC(CCNCC2C(O)=CC=CC=2)=C(OC)C=1	Blocker	Blocker	91.69	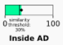	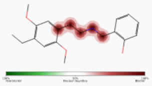	Moderate blocker	34.2	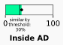	5.17	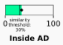	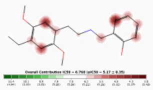

25B-NBOH

Target Name	ChEMBL-ID	UniProt ID	PDB Visualization	TTD ID	Probability	Model accuracy
DNA-(apurinic or apyrimidinic site) lyase	CHEMBL5619	P27695	6BOW	T13348	98.62%	91.11%
Cathepsin D	CHEMBL2581	P07339	4OD9	T67102	95.71%	98.95%
Nuclear factor NF-kappa-B p105 subunit	CHEMBL3251	P19838	1SVC	*Not Available*	95.43%	96.09%
Proteasome component C5	CHEMBL4208	P20618	6KWY	*Not Available*	92.98%	90%
Serotonin 2c (5-HT2c) receptor	CHEMBL225	P28335	6BQH	T83813	92.76%	89.62%
Glucose transporter	CHEMBL2535	P11166	6THA	*Not Available*	91.9%	98.75%
Transcription intermediary factor 1-alpha	CHEMBL3108638	O15164	4YBM	*Not Available*	90.89%	95.56%
Endoplasmic reticulum-associated amyloid beta-peptide-binding protein	CHEMBL4159	Q99714	2O23	*Not Available*	90.61%	70.16%
Kruppel-like factor 5	CHEMBL1293249	Q13887	*Not Available*	*Not Available*	90.45%	86.33%
Glycine transporter 2	CHEMBL3060	Q9Y345	*Not Available*	*Not Available*	88.41%	99.17%

Molecule 2

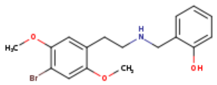

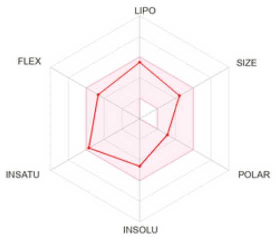

SMILES COc1cc(Br)c(cc1CCNCc1ccccc1O)OC

Physicochemical Properties	
Formula	C17H20BrNO3
Molecular weight	366.25 g/mol
Num. heavy atoms	22
Num. arom. heavy atoms	12
Fraction Csp3	0.29
Num. rotatable bonds	7
Num. H-bond acceptors	4
Num. H-bond donors	2
Molar Refractivity	91.02
TPSA	50.72 Å²

Lipophilicity	
Log $P_{o/w}$ (iLOGP)	3.56
Log $P_{o/w}$ (XLOGP3)	4.08
Log $P_{o/w}$ (WLOGP)	3.35
Log $P_{o/w}$ (MLOGP)	2.86
Log $P_{o/w}$ (SILICOS-IT)	4.08
Consensus Log $P_{o/w}$	3.58

Water Solubility	
Log *S* (ESOL)	-4.62
Solubility	8.73e-03 mg/ml ; 2.38e-05 mol/l
Class	Moderately soluble
Log *S* (Ali)	-4.85
Solubility	5.17e-03 mg/ml ; 1.41e-05 mol/l
Class	Moderately soluble
Log *S* (SILICOS-IT)	-6.66
Solubility	7.98e-05 mg/ml ; 2.18e-07 mol/l
Class	Poorly soluble

Pharmacokinetics	
GI absorption	High
BBB permeant	Yes
P-gp substrate	No
CYP1A2 inhibitor	Yes
CYP2C19 inhibitor	Yes
CYP2C9 inhibitor	Yes
CYP2D6 inhibitor	Yes
CYP3A4 inhibitor	Yes
Log K_p (skin permeation)	-5.64 cm/s

Druglikeness	
Lipinski	Yes; 0 violation
Ghose	Yes
Veber	Yes
Egan	Yes
Muegge	Yes
Bioavailability Score	0.55

Medicinal Chemistry	
PAINS	1 alert: mannich_A
Brenk	0 alert
Leadlikeness	No; 2 violations: MW>350, XLOGP3>3.5
Synthetic accessibility	2.36

25B-NBOH

Oral toxicity prediction results for input compound

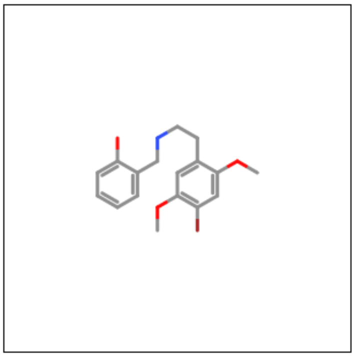

Predicted LD50: 400mg/kg

Predicted Toxicity Class: 4

1	2	3	4	5	6

Average similarity: 68.06%

Prediction accuracy: 68.07%

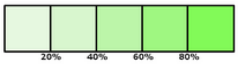

Name	COC1=CC(=C(C=C1CCN
Molweight	366.25
Number of hydrogen bond acceptors	4
Number of hydrogen bond donors	2
Number of atoms	22
Number of bonds	23
Number of rotable bonds	7
Molecular refractivity	91.02
Topological Polar Surface Area	50.72
octanol/water partition coefficient(logP)	3.9

Classification	Target	Shorthand	Prediction	Probability
Organ toxicity	Hepatotoxicity	dili	Inactive	0.74
Organ toxicity	Neurotoxicity	neuro	Active	0.57
Organ toxicity	Nephrotoxicity	nephro	Active	0.52
Organ toxicity	Respiratory toxicity	respi	Active	0.84
Organ toxicity	Cardiotoxicity	cardio	Inactive	0.57
Toxicity end points	Carcinogenicity	carcino	Inactive	0.61
Toxicity end points	Immunotoxicity	immuno	Active	0.99
Toxicity end points	Mutagenicity	mutagen	Inactive	0.69
Toxicity end points	Cytotoxicity	cyto	Inactive	0.67
Toxicity end points	BBB-barrier	bbb	Active	0.68
Toxicity end points	Ecotoxicity	eco	Active	0.51
Toxicity end points	Clinical toxicity	clinical	Active	0.55
Toxicity end points	Nutritional toxicity	nutri	Inactive	0.61
Tox21-Nuclear receptor signalling pathways	Aryl hydrocarbon Receptor (AhR)	nr_ahr	Inactive	0.94
Tox21-Nuclear receptor signalling pathways	Androgen Receptor (AR)	nr_ar	Inactive	0.96
Tox21-Nuclear receptor signalling pathways	Androgen Receptor Ligand Binding Domain (AR-LBD)	nr_ar_lbd	Inactive	0.95
Tox21-Nuclear receptor signalling pathways	Aromatase	nr_aromatase	Inactive	0.88
Tox21-Nuclear receptor signalling pathways	Estrogen Receptor Alpha (ER)	nr_er	Inactive	0.88
Tox21-Nuclear receptor signalling pathways	Estrogen Receptor Ligand Binding Domain (ER-LBD)	nr_er_lbd	Inactive	0.96
Tox21-Nuclear receptor signalling pathways	Peroxisome Proliferator Activated Receptor Gamma (PPAR-Gamma)	nr_ppar_gamma	Inactive	0.96
Tox21-Stress response pathways	Nuclear factor (erythroid-derived 2)-like 2/antioxidant responsive element (nrf2/ARE)	sr_are	Inactive	0.93
Tox21-Stress response pathways	Heat shock factor response element (HSE)	sr_hse	Inactive	0.93
Tox21-Stress response pathways	Mitochondrial Membrane Potential (MMP)	sr_mmp	Inactive	0.89
Tox21-Stress response pathways	Phosphoprotein (Tumor Supressor) p53	sr_p53	Inactive	0.90
Tox21-Stress response pathways	ATPase family AAA domain-containing protein 5 (ATAD5)	sr_atad5	Inactive	0.98
Molecular Initiating Events	Thyroid hormone receptor alpha (THRα)	mie_thr_alpha	Inactive	0.90
Molecular Initiating Events	Thyroid hormone receptor beta (THRβ)	mie_thr_beta	Inactive	0.78
Molecular Initiating Events	Transtyretrin (TTR)	mie_ttr	Inactive	0.97
Molecular Initiating Events	Ryanodine receptor (RYR)	mie_ryr	Inactive	0.98
Molecular Initiating Events	GABA receptor (GABAR)	mie_gabar	Inactive	0.96
Molecular Initiating Events	Glutamate N-methyl-D-aspartate receptor (NMDAR)	mie_nmdar	Inactive	0.92
Molecular Initiating Events	alpha-amino-3-hydroxy-5-methyl-4-isoxazolepropionate receptor (AMPAR)	mie_ampar	Inactive	0.97
Molecular Initiating Events	Kainate receptor (KAR)	mie_kar	Inactive	0.99
Molecular Initiating Events	Achetylcholinesterase (AChE)	mie_ache	Inactive	0.74
Molecular Initiating Events	Constitutive androstane receptor (CAR)	mie_car	Inactive	0.98
Molecular Initiating Events	Pregnane X receptor (PXR)	mie_pxr	Inactive	0.92
Molecular Initiating Events	NADH-quinone oxidoreductase (NADHOX)	mie_nadhox	Inactive	0.97
Molecular Initiating Events	Voltage gated sodium channel (VGSC)	mie_vgsc	Inactive	0.95
Molecular Initiating Events	Na+/I- symporter (NIS)	mie_nis	Inactive	0.98
Metabolism	Cytochrome CYP1A2	CYP1A2	Inactive	0.69
Metabolism	Cytochrome CYP2C19	CYP2C19	Active	0.53
Metabolism	Cytochrome CYP2C9	CYP2C9	Active	0.52
Metabolism	Cytochrome CYP2D6	CYP2D6	Active	0.79
Metabolism	Cytochrome CYP3A4	CYP3A4	Inactive	0.71
Metabolism	Cytochrome CYP2E1	CYP2E1	Inactive	0.96

25B-NBOH

000	Inhibit CYP2C19 Activity	Liver	0.983	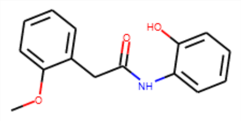
001	Differential cytotoxicity (isogenic chicken DT40 Rev3 mutant cell line)	Genotoxicity	0.987	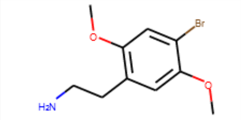
002	Modulator of Serotonin 2a (5-HT2a) receptor	Nervous system, blood, Heart	0.993	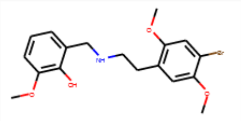
003	Differential cytotoxicity against isogenic chicken DT40 cell lines with known DNA damage response pathways - Rad54/ Ku70 mutant cell line	Genotoxicity	0.992	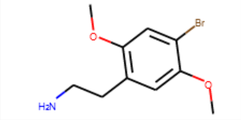
004	Modulator of Serotonin 2c (5-HT2c) receptor	Nervous system	0.999	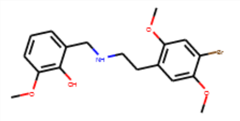
005	Differential cytotoxicity (isogenic chicken DT40 cell lines)	Genotoxicity	0.982	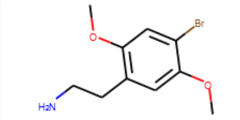
006	Antagonist of the thyroid receptor (TR) signaling pathway	Endocrine, Heart	0.983	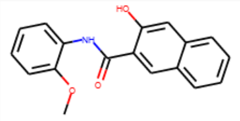
007	Modulator of Dopamine D1 receptor	Central nervous system, Kidney, Heart	0.984	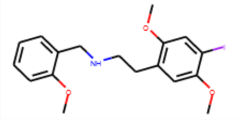

**Chemical Exposure**	**Molecular initiating event** *in chemico*	**Cellular response** *in vitro*		**Tissue / Organ response** *in vivo*	**Organism response** *in vivo*	**Pred-Skin 3.0 Outcome** *in silico*
•Skin Penetration •Electrophilic substance: directly or via auto-oxidation or metabolism	Covalent interaction with proteins in the skin (OECD442C) Haptenation: covalent modification of epidermal proteins	Keratinocyte responses (OECD442D) • Activation of inflammatory cytokines •Induce cytoprotective genes	Dendritic cells (DCs) (OECD442E) • Induction of inflammatory cytokines •Mobilization of DCs	Proliferation of antigen-specific T cells (OECD429) •Histocompatibility complex representation by DCs •Activation of T cells •Proliferation of activated T cells	Inflammation upon challenge allergen To maximise the use of existing knowledge, we also incorporate historical HRIPT (human repeated insult patch test) and HMT (human maximization test)	The Bayesian model is a consensus model integrating predictions from all the other assays for an integrative qualitative risk assessment (QRA) of skin sensitization based on the weight of evidence (WoE).
•Exposure consideration ? •Physicochemical and Biopharmaceutical properties ? •Skin Penetration ? •Skin Metabolism ? 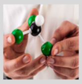	**Prediction DPRA** Sensitizer (+) (AD, Confiability) (Outside , 63.0%) Probability map 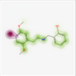	**Prediction KeratinoSens** Non-Sensitizer (-) (AD, Confiability) (Outside, 89.6%) Probability map 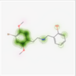	**Prediction h-CLAT** Non-Sensitizer (-) (AD, Confiability) (Outside, 64.0%) Probability map 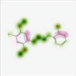	**Prediction LLNA** Non-Sensitizer (-) (AD, Confiability) (Outside, 60.0%) Probability map 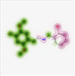	**Prediction HRIPT/HMT** Non-Sensitizer (-) (AD, Confiability) (Outside, 95.1%) Probability map 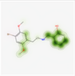	**Bayesian Outcome** Non-sensitizer (-) (Confiability) (High)

Low (-) confidence prediction for the Bayesian model means two or more individual predictions are in disagreement with Bayesian Outcome.

SMILES	Consensus Weighted	Binary Prediction	Confiabilty %	Applicability Domain	Fragment Contribution Maps and Explainable AI (XAI) for the Binary Model	Multiclass Prediction	Confiabilty %	Applicability Domain	Reg. prediction (pIC50)	Applicability Domain	Fragment Contribution Maps for the Regression Model
COC1C(CCNCC2C(O)=CC=CC=2)=CC(OC)=C(Br)C=1	Blocker	Blocker	97.29	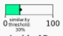 Inside AD	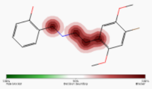	Moderate blocker	39.7	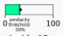 Inside AD	5.386	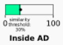 Inside AD	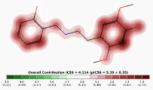

25I-NBOH

Target Name	ChEMBL-ID	UniProt ID	PDB Visualization	TTD ID	Probability	Model accuracy
DNA-(apurinic or apyrimidinic site) lyase	CHEMBL5619	P27695	6BOW	T13348	98.99%	91.11%
Nuclear factor NF-kappa-B p105 subunit	CHEMBL3251	P19838	1SVC	*Not Available*	95.02%	96.09%
Cathepsin D	CHEMBL2581	P07339	4OD9	T67102	93.16%	98.95%
Endoplasmic reticulum-associated amyloid beta-peptide-binding protein	CHEMBL4159	Q99714	2O23	*Not Available*	92.18%	70.16%
Proteasome component C5	CHEMBL4208	P20618	6KWY	*Not Available*	91.2%	90%
Kruppel-like factor 5	CHEMBL1293249	Q13887	*Not Available*	*Not Available*	90.04%	86.33%
Transcription intermediary factor 1-alpha	CHEMBL3108638	O15164	4YBM	*Not Available*	89.69%	95.56%
Glucose transporter	CHEMBL2535	P11166	6THA	*Not Available*	89.27%	98.75%
Tyrosyl-DNA phosphodiesterase 1	CHEMBL1075138	Q9NUW8	6N0D	*Not Available*	87.68%	71.22%
Glycine transporter 2	CHEMBL3060	Q9Y345	*Not Available*	*Not Available*	86.31%	99.17%

Molecule 3

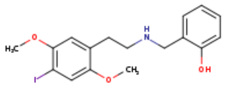

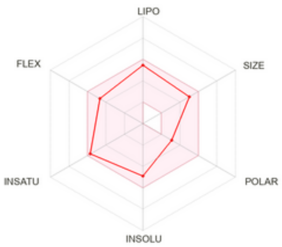

SMILES COc1cc(I)c(cc1CCNCc1ccccc1O)OC

Physicochemical Properties	
Formula	C17H20INO3
Molecular weight	413.25 g/mol
Num. heavy atoms	22
Num. arom. heavy atoms	12
Fraction Csp3	0.29
Num. rotatable bonds	7
Num. H-bond acceptors	4
Num. H-bond donors	2
Molar Refractivity	96.03
TPSA	50.72 Å²

Lipophilicity	
Log $P_{o/w}$ (iLOGP)	3.56
Log $P_{o/w}$ (XLOGP3)	4.04
Log $P_{o/w}$ (WLOGP)	3.19
Log $P_{o/w}$ (MLOGP)	2.97
Log $P_{o/w}$ (SILICOS-IT)	4.36
Consensus Log $P_{o/w}$	3.63

Water Solubility	
Log *S* (ESOL)	-4.89
Solubility	5.34e-03 mg/ml ; 1.29e-05 mol/l
Class	Moderately soluble
Log *S* (Ali)	-4.81
Solubility	6.42e-03 mg/ml ; 1.55e-05 mol/l
Class	Moderately soluble
Log *S* (SILICOS-IT)	-6.72
Solubility	7.86e-05 mg/ml ; 1.90e-07 mol/l
Class	Poorly soluble

Pharmacokinetics	
GI absorption	High
BBB permeant	Yes
P-gp substrate	No
CYP1A2 inhibitor	Yes
CYP2C19 inhibitor	Yes
CYP2C9 inhibitor	Yes
CYP2D6 inhibitor	Yes
CYP3A4 inhibitor	Yes
Log K_p (skin permeation)	-5.95 cm/s

Druglikeness	
Lipinski	Yes; 0 violation
Ghose	Yes
Veber	Yes
Egan	Yes
Muegge	Yes
Bioavailability Score	0.55

Medicinal Chemistry	
PAINS	1 alert: mannich_A
Brenk	1 alert: iodine
Leadlikeness	No; 2 violations: MW>350, XLOGP3>3.5
Synthetic accessibility	2.59

25I-NBOH

Oral toxicity prediction results for input compound

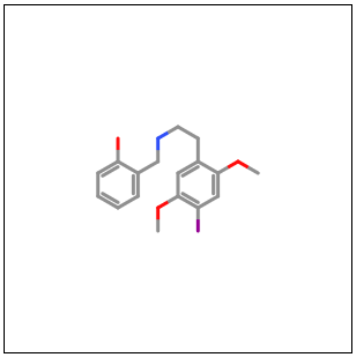

Predicted LD50: 300mg/kg

Predicted Toxicity Class: 3

1 2 3 4 5 6

Average similarity: 63.77%

Prediction accuracy: 68.07%

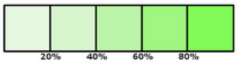

Name	COC1=CC(=C(C=C1CCN
Molweight	413.25
Number of hydrogen bond acceptors	4
Number of hydrogen bond donors	2
Number of atoms	22
Number of bonds	23
Number of rotable bonds	7
Molecular refractivity	96.03
Topological Polar Surface Area	50.72
octanol/water partition coefficient(logP)	3.74

Classification	Target	Shorthand	Prediction	Probability
Organ toxicity	Hepatotoxicity	dili	Inactive	0.74
Organ toxicity	Neurotoxicity	neuro	Active	0.56
Organ toxicity	Nephrotoxicity	nephro	Active	0.54
Organ toxicity	Respiratory toxicity	respi	Active	0.85
Organ toxicity	Cardiotoxicity	cardio	Inactive	0.56
Toxicity end points	Carcinogenicity	carcino	Inactive	0.66
Toxicity end points	Immunotoxicity	immuno	Active	0.99
Toxicity end points	Mutagenicity	mutagen	Inactive	0.68
Toxicity end points	Cytotoxicity	cyto	Inactive	0.70
Toxicity end points	BBB-barrier	bbb	Active	0.67
Toxicity end points	Ecotoxicity	eco	Active	0.50
Toxicity end points	Clinical toxicity	clinical	Active	0.55
Toxicity end points	Nutritional toxicity	nutri	Inactive	0.55
Tox21-Nuclear receptor signalling pathways	Aryl hydrocarbon Receptor (AhR)	nr_ahr	Inactive	0.94
Tox21-Nuclear receptor signalling pathways	Androgen Receptor (AR)	nr_ar	Inactive	0.96
Tox21-Nuclear receptor signalling pathways	Androgen Receptor Ligand Binding Domain (AR-LBD)	nr_ar_lbd	Inactive	0.97
Tox21-Nuclear receptor signalling pathways	Aromatase	nr_aromatase	Inactive	0.90
Tox21-Nuclear receptor signalling pathways	Estrogen Receptor Alpha (ER)	nr_er	Inactive	0.86
Tox21-Nuclear receptor signalling pathways	Estrogen Receptor Ligand Binding Domain (ER-LBD)	nr_er_lbd	Inactive	0.96
Tox21-Nuclear receptor signalling pathways	Peroxisome Proliferator Activated Receptor Gamma (PPAR-Gamma)	nr_ppar_gamma	Inactive	0.95
Tox21-Stress response pathways	Nuclear factor (erythroid-derived 2)-like 2/antioxidant responsive element (nrf2/ARE)	sr_are	Inactive	0.94
Tox21-Stress response pathways	Heat shock factor response element (HSE)	sr_hse	Inactive	0.94
Tox21-Stress response pathways	Mitochondrial Membrane Potential (MMP)	sr_mmp	Inactive	0.88
Tox21-Stress response pathways	Phosphoprotein (Tumor Supressor) p53	sr_p53	Inactive	0.92
Tox21-Stress response pathways	ATPase family AAA domain-containing protein 5 (ATAD5)	sr_atad5	Inactive	0.98
Molecular Initiating Events	Thyroid hormone receptor alpha (THRα)	mie_thr_alpha	Inactive	0.90
Molecular Initiating Events	Thyroid hormone receptor beta (THRβ)	mie_thr_beta	Inactive	0.78
Molecular Initiating Events	Transtyretrin (TTR)	mie_ttr	Inactive	0.97
Molecular Initiating Events	Ryanodine receptor (RYR)	mie_ryr	Inactive	0.98
Molecular Initiating Events	GABA receptor (GABAR)	mie_gabar	Inactive	0.96
Molecular Initiating Events	Glutamate N-methyl-D-aspartate receptor (NMDAR)	mie_nmdar	Inactive	0.92
Molecular Initiating Events	alpha-amino-3-hydroxy-5-methyl-4-isoxazolepropionate receptor (AMPAR)	mie_ampar	Inactive	0.97
Molecular Initiating Events	Kainate receptor (KAR)	mie_kar	Inactive	0.99
Molecular Initiating Events	Achetylcholinesterase (AChE)	mie_ache	Inactive	0.74
Molecular Initiating Events	Constitutive androstane receptor (CAR)	mie_car	Inactive	0.98
Molecular Initiating Events	Pregnane X receptor (PXR)	mie_pxr	Inactive	0.92
Molecular Initiating Events	NADH-quinone oxidoreductase (NADHOX)	mie_nadhox	Inactive	0.97
Molecular Initiating Events	Voltage gated sodium channel (VGSC)	mie_vgsc	Inactive	0.95
Molecular Initiating Events	Na+/I- symporter (NIS)	mie_nis	Inactive	0.98
Metabolism	Cytochrome CYP1A2	CYP1A2	Inactive	0.68
Metabolism	Cytochrome CYP2C19	CYP2C19	Active	0.50
Metabolism	Cytochrome CYP2C9	CYP2C9	Active	0.51
Metabolism	Cytochrome CYP2D6	CYP2D6	Active	0.79
Metabolism	Cytochrome CYP3A4	CYP3A4	Inactive	0.71
Metabolism	Cytochrome CYP2E1	CYP2E1	Inactive	0.96

25I-NBOH

Index	Action	Injury	Confidence	Similar active compound in database
000	Differential cytotoxicity against isogenic chicken DT40 cell lines with known DNA damage response pathways - Rad54/ Ku70 mutant cell line	Genotoxicity	0.992	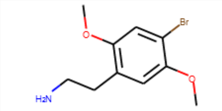
001	Differential cytotoxicity (isogenic chicken DT40 cell lines)	Genotoxicity	0.985	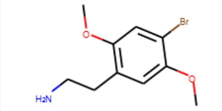
002	Modulator of Serotonin 2c (5-HT2c) receptor	Nervous system	0.994	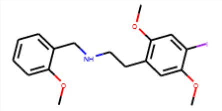
003	Modulator of Dopamine D1 receptor	Central nervous system, Kidney, Heart	0.984	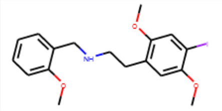
004	Modulator of Serotonin 2a (5-HT2a) receptor	Nervous system, blood, Heart	0.992	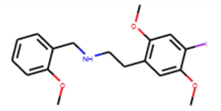
005	Differential cytotoxicity (isogenic chicken DT40 Rev3 mutant cell line)	Genotoxicity	0.988	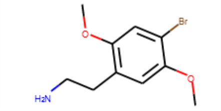
006	Antagonist of the thyroid receptor (TR) signaling pathway	Endocrine, Heart	0.984	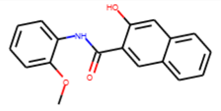

**Chemical Exposure**	**Molecular initiating event** *in chemico*	**Cellular response** *in vitro*		**Tissue / Organ response** *in vivo*	**Organism response** *in vivo*	**Pred-Skin 3.0 Outcome** *in silico*
•Skin Penetration •Electrophilic substance: directly or via auto-oxidation or metabolism	Covalent interaction with proteins in the skin (OECD442C) Haptenation: covalent modification of epidermal proteins	Keratinocyte responses (OECD442D) • Activation of inflammatory cytokines •Induce cytoprotective genes	Dendritic cells (DCs) (OECD442E) • Induction of inflammatory cytokines •Mobilization of DCs	Proliferation of antigen-specific T cells (OECD429) •Histocompatibility complex representation by DCs •Activation of T cells •Proliferation of activated T cells	Inflammation upon challenge allergen To maximise the use of existing knowledge, we also incorporate historical HRIPT (human repeated insult patch test) and HMT (human maximization test)	The Bayesian model is a consensus model integrating predictions from all the other assays for an integrative qualitative risk assessment (QRA) of skin sensitization based on the weight of evidence (WoE).
•Exposure consideration ? •Physicochemical and Biopharmaceutical properties ? •Skin Penetration ? •Skin Metabolism ? 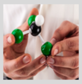	**Prediction DPRA** Sensitizer (+) (AD, Confiability) (Outside , 59.5%) Probability map 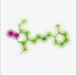	**Prediction KeratinoSens** Sensitizer (+) (AD, Confiability) (Outside, 90.0%) Probability map 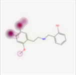	**Prediction h-CLAT** Non-Sensitizer (-) (AD, Confiability) (Outside, 63.7%) Probability map 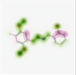	**Prediction LLNA** Non-Sensitizer (-) (AD, Confiability) (Outside, 60.0%) Probability map 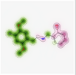	**Prediction HRIPT/HMT** Non-Sensitizer (-) (AD, Confiability) (Outside, 96.4%) Probability map 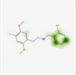	**Bayesian Outcome** Non-sensitizer (-) (Confiability) (High)

Low (-) confidence prediction for the Bayesian model means two or more individual predictions are in disagreement with Bayesian Outcome.

SMILES	Consensus Weighted	Binary Prediction	Confiabilty %	Applicability Domain	Fragment Contribution Maps and Explainable AI (XAI) for the Binary Model	Multiclass Prediction	Confiabilty %	Applicability Domain	Reg. prediction (pIC50)	Applicability Domain	Fragment Contribution Maps for the Regression Model
COC1C(CCNCC2C(O)=CC=CC=2)=CC(OC)=C(I)C=1	Blocker	Blocker	93.19	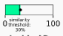 0 100 Inside AD	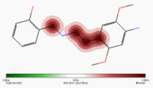	Moderate blocker	37.57	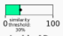 0 100 Inside AD	5.331	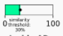 0 100 Inside AD	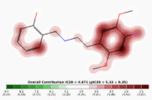
